# Heterogeneity of Treatment Effect for Intra‐Aortic Balloon Pump Use Across Lactate Trajectories in Cardiogenic Shock Patients Supported by VA‐ECMO: An Analysis of the Chinese Extracorporeal Life Support Registry

**DOI:** 10.1002/mco2.70698

**Published:** 2026-03-18

**Authors:** Xiang‐Jie Duan, Bo Wang, Cheng‐Long Li, Wan Chen, Peng Ding, Jie‐Lian Zhu, Jun‐Wei Wang, Xiao‐Tong Hou, Hai‐Yan Yin, Wan‐Jie Gu

**Affiliations:** ^1^ Department of Intensive Care Unit The First Affiliated Hospital of Jinan University Guangzhou China; ^2^ Medical Education Center of Jinan University Guangzhou China; ^3^ Department of Emergency Medicine, Research Center of Cardiovascular Disease, the People's Hospital of Guangxi Zhuang Autonomous Region Guangxi Academy of Medical Sciences Nanning China; ^4^ Center For Cardiac Intensive Care, Beijing Anzhen Hospital Capital Medical University Beijing China

**Keywords:** cardiogenic shock, heterogeneity of treatment effect, intra‐aortic balloon pump, lactate trajectory, myocardial infarction, veno‐arterial extracorporeal membrane oxygenation

## Abstract

This study aimed to investigate the heterogeneity of treatment effect for intra‐aortic balloon pump (IABP) across various lactate trajectories in patients with acute myocardial infarction‐related cardiogenic shock (AMICS) supported by veno‐arterial extracorporeal membrane oxygenation (VA‐ECMO). Retrospective data from the China Extracorporeal Life Support Registry included AMICS patients who received VA‐ECMO. The latent class growth model was used to identify distinct lactate trajectories. The primary outcome was in‐hospital mortality. Baseline characteristics and outcomes were compared across trajectory classes, and the heterogeneity of treatment effect for IABP was assessed. Among 1264 patients, three lactate trajectories were identified. Compared with Class 1, both Class 2 (odds ratio [OR] 2.03, 95% confidence interval [CI] 1.54–2.67) and Class 3 (OR 3.99, 95% CI 2.77–5.78) had significantly higher in‐hospital mortality. Moreover, heterogeneity of treatment effect across the classes was found. IABP use was associated with increased risks of in‐hospital mortality and multiple complications (bleeding, renal, metabolic, and infection) in Class 1, whereas no associations were observed in Class 2 or Class 3, except for a higher risk of infection in Class 2. In summary, lactate trajectories can stratify mortality risk in AMICS patients receiving VA‐ECMO support and reflect heterogeneous responses to IABP treatment.

## Graphical abstract

Lactate trajectories can stratify the mortality risk in patients with acute myocardial infarction‐related cardiogenic shock receiving VA‐ECMO and reflect heterogeneity of treatment effect for intra‐aortic balloon pump.

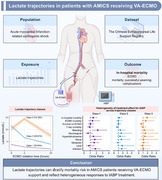

## 1 Introduction

Cardiogenic shock (CS) remains one of the most severe complications of acute myocardial infarction, with reported mortality rates ranging from 47.9% to 77.7% [1, 2]. Despite initial medical therapy, many patients progress to refractory CS and require veno‐arterial extracorporeal membrane oxygenation (VA‐ECMO), which has become the most commonly implemented device of temporary mechanical circulatory support in this context. Despite its expanded use, overall survival has not substantially improved [3, 4, 5]. The efficacy of VA‐ECMO is influenced by multiple factors, including concomitant therapies, patient selection, and organ perfusion status during support [6, 7].

Among these factors, the clinical benefit of combining VA‐ECMO with intra‐aortic balloon pump (IABP) remains controversial [7, 8, 9, 10, 11, 12]. The IABP‐SHOCK II trial demonstrated no survival advantage with routine IABP use in acute myocardial infarction‐related cardiogenic shock (AMICS) [8, 9], and current European Society of Cardiology (ESC) guidelines recommend against its routine application (Class III B) [13, 14, 15]. Despite this, the device continues to be widely used, suggesting that certain patient subgroups may still derive benefit [16]. This variability likely reflects substantial heterogeneity in patients’ pathophysiological profiles, including left ventricular function, systemic vascular resistance, and aortic compliance, which may influence the hemodynamic response to IABP. However, reliable dynamic markers capable of objectively identifying patients who may benefit from IABP during VA‐ECMO support remain lacking.

Lactate is an established metabolic biomarker of tissue hypoperfusion, and both absolute levels and dynamic changes, particularly lactate clearance, are strongly associated with patient outcomes [17, 18, 19, 20, 21]. Compared with single time‐point measurements, lactate trajectories provide a more dynamic assessment of disease progression and resuscitation response, and have been increasingly applied in critically ill patients [22, 23, 24, 25, 26]. Among patients with CS receiving VA‐ECMO, changes in lactate trajectories may indicate improved organ perfusion or persistent shock [17]. However, evidence specific to the AMICS population—which constitutes the largest etiology of CS—remains scarce. Only one post hoc analysis has characterized daily lactate trajectories during early ECMO support, and that study was neither restricted to AMICS nor designed to evaluate treatment effect modification by IABP [27]. In the absence of dynamic and objective markers to assess whether IABP improves organ perfusion in AMICS, lactate trajectories, which reflect systemic perfusion and metabolic recovery, may serve as a clinically relevant tool for phenotyping patients and identifying those most likely to benefit from IABP.

Therefore, this study aimed to (1) identify distinct lactate trajectories in patients with AMICS supported by VA‐ECMO using latent class growth modeling (LCGM); (2) compare clinical characteristics and outcomes across these trajectory classes; and (3) evaluate the heterogeneity of treatment effect for IABP use across trajectory classes.

## 2 Results

### 2.1 Patient Characteristics

**FIGURE 1:**
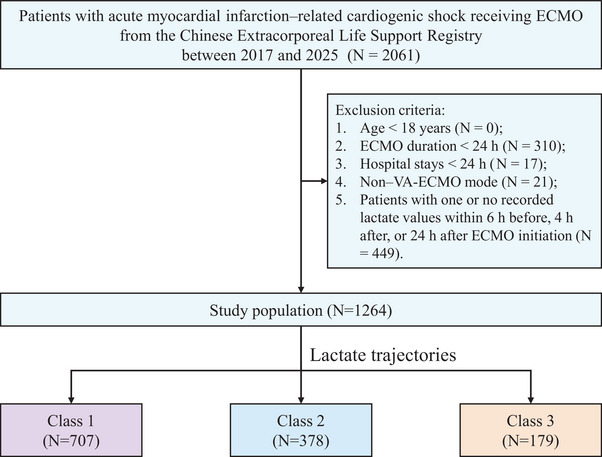
Study flow diagram of patients included in the analysis. VA‐ECMO, veno‐arterial extracorporeal membrane oxygenation.

The screening process for patients with AMICS is shown in Figure 1. A total of 1264 patients with AMICS were included. The mean age was 60.09 ± 11.32 years, and 1034 (81.8%) were male. The rate of IABP use was 44.6% (564/1264). The overall in‐hospital mortality, ECMO mortality, ECMO successful weaning, and 7‐day mortality were 41.5%, 11.6%, 71.1%, and 16.7%, respectively. Other clinical characteristics are presented in Table 1.

**TABLE 1:** Clinical characteristics of patients with AMICS stratified by classes.

Characteristics	Overall (*N* = 1264)	Class 1 (*N* = 707)	Class 2 (*N* = 378)	Class 3 (*N* = 179)	*p* value
Age (year)	60.09 (11.32)	59.48 (11.35)	60.37 (11.48)	61.91 (10.72)	0.026
Sex, *n* (%)					0.229
Female	1034 (81.8%)	589 (83.3%)	305 (80.7%)	140 (78.2%)	
Male	230 (18.2%)	118 (16.7%)	73 (19.3%)	39 (21.8%)	
BMI (kg/m^2^)	24.1 (2.5)	24.1 (2.5)	24.1 (2.6)	24.0 (2.4)	0.755
Medical history, *n* (%)					
Cardiac surgery	27 (2.1%)	14 (2.0%)	8 (2.1%)	5 (2.8%)	0.797
Cardiac intervention	262 (20.7%)	137 (19.4%)	71 (18.8%)	54 (30.2%)	0.003
Myocardial infarction	246 (19.5%)	121 (17.1%)	75 (19.8%)	50 (27.9%)	0.005
Hypertension	655 (51.8%)	354 (50.1%)	206 (54.5%)	95 (53.1%)	0.356
Diabetes mellitus	405 (32.0%)	219 (31.0%)	120 (31.7%)	66 (36.9%)	0.316
Hyperlipidemia	198 (15.7%)	99 (14.0%)	67 (17.7%)	32 (17.9%)	0.187
Heart failure	178 (14.1%)	80 (11.3%)	66 (17.5%)	32 (17.9%)	0.006
Chronic respiratory diseases	46 (3.6%)	32 (4.5%)	11 (2.9%)	3 (1.7%)	0.127
Chronic kidney disease	57 (4.5%)	33 (4.7%)	11 (2.9%)	13 (7.3%)	0.066
Neurological disease	116 (9.2%)	63 (8.9%)	23 (6.1%)	30 (16.8%)	< 0.001
Anticoagulants	137 (10.8%)	54 (7.6%)	58 (15.3%)	25 (14.0%)	< 0.001
Smoking	561 (44.4%)	311 (44.0%)	177 (46.8%)	73 (40.8%)	0.387
Pre‐ECMO cardiac arrest, *n* (%)	349 (27.6%)	142 (20.1%)	145 (38.4%)	62 (34.6%)	< 0.001
IABP use, *n* (%)	564 (44.6%)	309 (43.7%)	166 (43.9%)	89 (49.7%)	0.333
ECMO initiation time (h)	1.39 (−1.82, 20.68)	1.00 (−1.97, 20.60)	0.86 (−2.25, 12.50)	7.33 (0.08, 34.05)	< 0.001
Pre‐ECMO support, *n* (%)					
Mechanical ventilation	953 (75.4%)	479 (67.8%)	312 (82.5%)	162 (90.5%)	< 0.001
Vasopressors use	970 (76.7%)	511 (72.3%)	319 (84.4%)	140 (78.2%)	< 0.001
One type	419 (33.1%)	275 (38.9%)	113 (29.9%)	31 (17.3%)	
Two types	377 (29.8%)	175 (24.8%)	129 (34.1%)	73 (40.8%)	
Three types	174 (13.8%)	61 (8.6%)	77 (20.4%)	36 (20.1%)	
Vasoactive‐inotropic score	38.75 (4.00, 102.69)	22.84 (0.00, 86.30)	57.50 (12.00, 128.00)	62.33 (13.80, 150.00)	< 0.001
Pre‐ECMO hemodynamics					
Heart rate (beats/min)	102 (65, 127)	102 (69, 125)	102 (52, 134)	96 (50, 125)	0.381
SBP (mmHg)	73 (60, 85)	78 (63, 87)	70 (50, 80)	69 (50, 79)	< 0.001
DBP (mmHg)	45 (34, 54)	49 (39, 56)	40 (30, 50)	40 (30, 50)	< 0.001
MAP (mmHg)	54 (43, 64)	58 (47, 67)	50 (37, 60)	51 (36, 60)	< 0.001
Pre‐ECMO blood gases					
pH	7.27 (0.15)	7.32 (0.11)	7.17 (0.15)	7.23 (0.16)	< 0.001
HCO_3_^−^ (mmol/L)	17.57 (14.10, 20.30)	19.00 (16.50, 21.60)	14.30 (11.30, 17.80)	15.90 (12.40, 19.40)	< 0.001
PaO_2_ (mmHg)	82.40 (65.00, 116.85)	85.40 (67.00, 113.00)	78.50 (60.90, 112.19)	87.50 (62.00, 138.00)	0.03
PaCO_2_ (mmHg)	35.61 (30.00, 43.00)	35.00 (30.00, 40.97)	37.00 (29.50, 46.00)	36.00 (29.16, 46.00)	0.02
Lactate (mmol/L)	7.07 (4.32)	4.30 (1.87)	10.87 (3.21)	10.04 (5.18)	< 0.001
SpO_2_ (%)	89.25(11.16)	90.73 (9.63)	87.18 (12.00)	87.75 (13.80)	< 0.001
Blood gases for 4 h during ECMO					
pH	7.37 (0.10)	7.39 (0.08)	7.34 (0.11)	7.31 (0.12)	< 0.001
HCO_3_^−^ (mmol/L)	20.50 (17.90, 23.00)	21.13 (18.80, 23.45)	20.00 (17.40, 22.20)	18.00 (14.80, 21.10)	< 0.001
PaO_2_ (mmHg)	144.07 (90.15, 269.50)	138.84 (91.20, 240.00)	138.00 (90.00, 285.00)	217.00 (85.00, 349.00)	0.003
PaCO_2_ (mmHg)	35.00 (29.40, 40.60)	34.40 (29.00, 40.00)	36.00 (30.40, 42.00)	34.00 (28.60, 41.90)	0.002
Lactate (mmol/L)	3.90 (2.20, 7.65)	2.50 (1.70, 3.70)	6.51 (4.30, 8.60)	14.00 (11.30, 15.00)	< 0.001
SpO_2_ (%)	97.26 (4.51)	97.40 (4.17)	97.14 (4.68)	96.94 (5.36)	0.304
Blood gases for 24 h during ECMO					
pH	7.42 (0.08)	7.43 (0.07)	7.40 (0.08)	7.41 (0.10)	< 0.001
HCO_3_^−^ (mmol/L)	23.70 (21.10, 26.00)	23.80 (21.70, 26.00)	23.10 (20.90, 26.00)	24.00 (21.00, 28.00)	0.027
PaO_2_ (mmHg)	123.00 (91.00, 185.00)	121.00 (90.00, 168.00)	116.00 (91.00, 174.00)	154.00 (100.00, 247.00)	< 0.001
PaCO_2_ (mmHg)	36.00 (32.00, 41.00)	36.00 (31.90, 40.00)	36.53 (32.80, 43.00)	37.00 (32.00, 42.00)	0.021
Lactate (mmol/L)	2.20 (1.50, 3.67)	1.71 (1.30, 2.40)	3.06 (1.90, 5.00)	5.00 (2.70, 9.56)	< 0.001
SpO_2_ (%)	97.61 (3.45)	97.57 (3.22)	97.38 (4.12)	98.25 (2.63)	0.004
During ECMO support, *n* (%)					
Mechanical ventilation	1001 (79.2%)	503 (71.1%)	332 (87.8%)	166 (92.7%)	< 0.001
Vasopressors use	1041 (82.4%)	548 (77.5%)	340 (89.9%)	153 (85.5%)	< 0.001
One type	481 (38.1%)	302 (42.7%)	138 (36.5%)	41 (22.9%)	
Two types	418 (33.1%)	197 (27.9%)	143 (37.8%)	78 (43.6%)	
Three types	142 (11.2%)	49 (6.9%)	59 (15.6%)	34 (19.0%)	
Vasoactive‐inotropic score	20.00 (4.00, 60.00)	14.00 (2.00, 42.00)	27.50 (8.00, 75.00)	44.80 (8.00, 105.37)	< 0.001
ECMO assistance (h)	119.00 (72.00, 189.50)	118.73 (73.88, 187.00)	128.70 (77.67, 203.00)	99.50 (49.17, 167.72)	0.003
Length of hospital stay (days)	14.01 (7.90, 22.67)	15.50 (10.00, 23.00)	13.47 (6.00, 24.00)	10.00 (3.49, 18.17)	< 0.001
Length of ICU stay (days)	10.63 (6.00, 16.74)	10.90 (6.98, 17.00)	11.05 (5.60, 16.84)	8.23 (3.34, 13.50)	< 0.001
In‐hospital mortality, *n* (%)	524 (41.5%)	216 (30.6%)	187 (49.5%)	121 (67.6%)	< 0.001
ECMO mortality, *n* (%)	146 (11.6%)	49 (6.9%)	50 (13.2%)	47 (26.3%)	< 0.001
ECMO successful weaning, *n* (%)	899 (71.1%)	570 (80.6%)	248 (65.6%)	81 (45.3%)	< 0.001
7‐Day mortality, *n* (%)	211 (16.7%)	69 (9.8%)	84 (22.2%)	58 (32.4%)	<0.001
Complications, *n* (%)					
Bleeding	180 (14.2%)	75 (10.6%)	76 (20.1%)	29 (16.2%)	< 0.001
Neurological	79 (6.3%)	33 (4.7%)	27 (7.1%)	19 (10.6%)	0.009
Renal	1109 (87.7%)	602 (85.1%)	341 (90.2%)	166 (92.7%)	0.005
Metabolic	416 (32.9%)	176 (24.9%)	157 (41.5%)	83 (46.4%)	< 0.001
Limb	80 (6.3%)	34 (4.8%)	27 (7.1%)	19 (10.6%)	0.013
Hyperbilirubinemia	142 (11.2%)	51 (7.2%)	53 (14.0%)	38 (21.2%)	< 0.001
Infection	367 (29.0%)	199 (28.1%)	131 (34.7%)	37 (20.7%)	0.002

*Note*: Continuous data are expressed as mean (SD) or median (IQR). ECMO initiation time is defined by the interval between hospital admission and ECMO initiation.

Abbreviations: AMICS, acute myocardial infarction‐related cardiogenic shock. BMI, body mass index; DBP, diastolic blood pressure; ECMO, extracorporeal membrane oxygenation; IABP, intra‐aortic balloon pump; ICU, intensive care unit; IQR, interquartile range; MAP, mean arterial pressure; SBP, systolic blood pressure; SD, standard deviation.

A total of 449 patients were excluded due to missing lactate measurements at two or more of the three predefined time points. Compared with included patients, excluded patients showed no significant differences in age, sex, or body mass index. However, they had a lower prevalence of comorbidities and a lower rate of organ support therapy, suggesting a relatively milder clinical condition. Detailed comparisons are presented in Table S1.

### 2.2 Lactate Trajectory Classes

Among the predefined models with two to six potential classes, the model with K = 3 showed the lowest Bayesian information criterion (BIC), with entropy values all greater than 0.7, and the smallest subgroup size exceeding 10% of the total sample (Tables S2 and S3). The average posterior probabilities for all three classes were >0.7 (Table S4), and the fixed‐effect estimates of the three‐class longitudinal model are presented in Table S5. Considering statistical indicators, trajectory parsimony, and clinical interpretability, *K* = 3 was selected as the optimal trajectory model.

**FIGURE 2:**
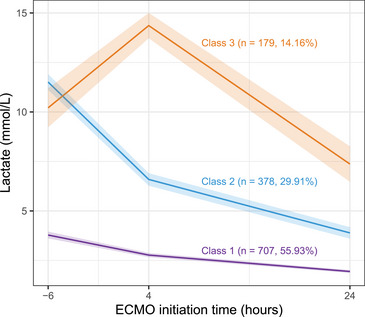
Lactate trajectory in the acute myocardial infarction‐related cardiogenic shock patients. Using latent class growth modeling, three lactate trajectory classes were identified. The means and 95% confidence intervals of class measurements for early lactate dynamics before and after ECMO initiation are presented. ECMO, extracorporeal membrane oxygenation.

Among the three classes, Class 1 accounted for 55.9% (707/1264) and was characterized by a mildly elevated lactate level followed by a slow decline, defined as the *“low–slow decline”* phenotype. Class 2 accounted for 29.9% (378/1264) and was characterized by a severely elevated lactate level followed by a rapid decline, defined as the “high–rapid decline” phenotype. Class 3 accounted for 14.2% (179/1264) and was characterized by a severely elevated lactate level with an initial sustained rise before a rapid decline, defined as the “high–delayed decline” phenotype. The trajectory curves of the three classes are shown in Figure 2.

### 2.3 Clinical Characteristics of Different Lactate Trajectory Classes

Baseline characteristics of the three clinical classes are presented in Table 1. Class 1: Patients were younger and had the lowest proportions of anticoagulant, heart failure, and myocardial infarction. The rates of pre‐ECMO cardiac arrest, mechanical ventilation, and vasoactive drug use were also the lowest. Their pre‐ECMO blood pressures were higher, indicating milder illness severity. Class 2: Patients had the earliest ECMO initiation and the longest duration of support, as well as the longest duration of mechanical ventilation. The proportions of pre‐ECMO cardiac arrest and vasoactive drug use were the highest, accompanied by the poorest arterial blood gas parameters. Class 3: Patients were the oldest and had the highest prevalence of heart failure, myocardial infarction, and neurological diseases. They exhibited the highest rates of mechanical ventilation and had the highest vasoactive‐inotropic scores before and after ECMO initiation. ECMO was initiated the latest and maintained for the shortest duration, indicating the most severe condition and poorest prognosis (Table 1).

Among the three trajectory classes, Class 1 showed the best prognosis, while Class 3 had the worst. Specifically, in‐hospital mortality rates were 30.6%, 49.5%, and 67.6% for Class 1, Class 2, and Class 3, respectively. Regarding ECMO‐related complications, except for infectious complications, patients in Class 1 had the lowest incidence of complications (*p* < 0.05), whereas patients in Class 3 had the highest incidence of neurological, renal, metabolic, limb, and hyperbilirubinemia complications (*p* < 0.05) (Table 1).

### 2.4 Association Between Lactate Trajectory Classes and Outcomes

#### 2.4.1 Primary Outcome

In the fully adjusted logistic regression model, using Class 1 as the reference, both Class 2 (odds ratio [OR] 2.03, 95% confidence interval [CI] 1.54–2.67, *p* < 0.001) and Class 3 (OR 3.99, 95% CI 2.77–5.78, *p* < 0.001) were associated with significantly higher risks of in‐hospital mortality. These findings were consistent with those of the unadjusted and partially adjusted models (Table 2), and similar results were observed in the subgroup analyses (Table S6).

**TABLE 2:** Association between classes and outcomes in patients with AMICS.

Outcomes	Class	Model 1		Model 2		Model 3	
		OR/HR (95% CI)	*p* value	OR/HR (95% CI)	*p* value	OR/HR (95% CI)	*p* value
In‐hospital mortality	Class 1	Reference		Reference		Reference	
	Class 2	2.23 (1.72, 2.88)	< 0.001	2.20 (1.70, 2.85)	< 0.001	2.03 (1.54, 2.67)	< 0.001
	Class 3	4.74 (3.35, 6.78)	< 0.001	4.58 (3.23, 6.56)	< 0.001	3.99 (2.77, 5.78)	< 0.001
ECMO mortality	Class 1	Reference		Reference		Reference	
	Class 2	2.05 (1.35, 3.11)	< 0.001	2.04 (1.34, 3.10)	< 0.001	1.85 (1.20, 2.87)	0.006
	Class 3	4.78 (3.07, 7.45)	< 0.001	4.73 (3.03, 7.37)	< 0.001	4.29 (2.70, 6.84)	< 0.001
ECMO successful weaning	Class 1	Reference		Reference		Reference	
	Class 2	0.46 (0.35, 0.61)	< 0.001	0.46 (0.35, 0.62)	< 0.001	0.49 (0.36, 0.66)	< 0.001
	Class 3	0.20 (0.14, 0.28)	< 0.001	0.21 (0.14, 0.29)	< 0.001	0.23 (0.16, 0.33)	< 0.001
7‐Day mortality	Class 1	Reference		Reference		Reference	
	Class 2	2.47 (1.80, 3.40)	< 0.001	2.40 (1.75, 3.31)	< 0.001	2.15 (1.54, 3.00)	< 0.001
	Class 3	3.88 (2.74, 5.50)	< 0.001	3.65 (2.57, 5.19)	< 0.001	3.22 (2.24, 4.63)	< 0.001
Bleeding	Class 1	Reference		Reference		Reference	
	Class 2	2.12 (1.50, 3.00)	< 0.001	2.10 (1.48, 2.98)	< 0.001	1.89 (1.31, 2.73)	< 0.001
	Class 3	1.63 (1.01, 2.57)	0.039	1.59 (0.99, 2.51)	0.051	1.49 (0.91, 2.40)	0.105
Neurological	Class 1	Reference		Reference		Reference	
	Class 2	1.57 (0.92, 2.65)	0.091	1.56 (0.92, 2.64)	0.096	1.28 (0.73, 2.23)	0.377
	Class 3	2.43 (1.32, 4.33)	0.003	2.40 (1.31, 4.30)	0.004	1.94 (1.02, 3.60)	0.038
Renal	Class 1	Reference		Reference		Reference	
	Class 2	1.61 (1.09, 2.42)	0.019	1.61 (1.09, 2.43)	0.019	1.33 (0.87, 2.06)	0.195
	Class 3	2.23 (1.26, 4.25)	0.009	2.25 (1.28, 4.30)	0.008	1.93 (1.05, 3.82)	0.044
Metabolic	Class 1	Reference		Reference		Reference	
	Class 2	2.14 (1.64, 2.80)	< 0.001	2.13 (1.64, 2.79)	< 0.001	2.08 (1.58, 2.74)	< 0.001
	Class 3	2.61 (1.86, 3.66)	< 0.001	2.57 (1.83, 3.62)	< 0.001	2.60 (1.83, 3.70)	< 0.001
Limb	Class 1	Reference		Reference		Reference	
	Class 2	1.52 (0.90, 2.56)	0.114	1.49 (0.87, 2.50)	0.138	1.43 (0.81, 2.48)	0.210
	Class 3	2.35 (1.28, 4.19)	0.004	2.22 (1.21, 3.96)	0.008	2.31 (1.21, 4.32)	0.009
Hyperbilirubinemia	Class 1	Reference		Reference		Reference	
	Class 2	2.10 (1.40, 3.16)	< 0.001	2.07 (1.38, 3.12)	< 0.001	2.29 (1.49, 3.51)	< 0.001
	Class 3	3.47 (2.18, 5.47)	< 0.001	3.35 (2.10, 5.29)	< 0.001	3.58 (2.21, 5.80)	< 0.001
Infection	Class 1	Reference		Reference		Reference	
	Class 2	1.35 (1.03, 1.77)	0.027	1.38 (1.05, 1.80)	0.020	1.35 (1.01, 1.81)	0.041
	Class 3	0.67 (0.44, 0.98)	0.044	0.68 (0.45, 1.01)	0.059	0.70 (0.46, 1.06)	0.103

*Note*: Model 1: unadjusted. Model 2: adjusted for age and sex. Model 3: adjusted for age, sex, BMI, smoking, cardiac surgery, cardiac intervention, anticoagulants, hypertension, diabetes mellitus, heart failure, myocardial infarction, hyperlipidemia, chronic respiratory diseases, neurological disease, chronic kidney disease, pre‐ECMO cardiac arrest, pre‐ECMO mechanical ventilation, and pre‐ECMO vasopressors. 7‐day mortality was analyzed using a Cox regression model (HR with 95% CI), and all other outcomes were analyzed using logistic regression models (OR with 95% CI).

Abbreviations: AMICS, acute myocardial infarction‐related cardiogenic shock; BMI, body mass index; CI, confidence interval; ECMO, extracorporeal membrane oxygenation; HR, hazard ratio; OR, odds ratio.

#### 2.4.2 Secondary Outcomes

**FIGURE 3:**
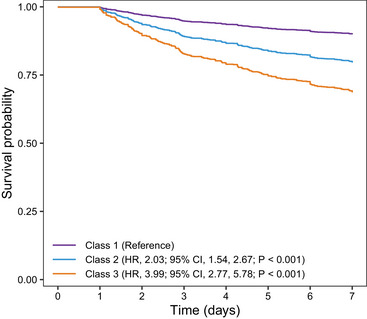
Adjusted Kaplan–Meier curve for 7‐day mortality according to lactate trajectory classes. HR, hazard ratio; CI, confidence interval.

Figure 3 presents the adjusted Kaplan–Meier survival curves for 7‐day mortality across the three classes. In the fully adjusted Cox regression model, using Class 1 as the reference, both Class 2 (hazard ratio [HR] 2.15, 95% CI 1.54–3.00, *p* < 0.001) and Class 3 (HR 3.22, 95% CI 2.24–4.63, *p* < 0.001) demonstrated significantly increased risks of 7‐day mortality. These results were consistent with those of the unadjusted and partially adjusted models (Table 2). ECMO mortality and ECMO successful weaning also showed a similar trend across all three models, with both Class 2 and Class 3 associated with increased ECMO mortality and lower ECMO successful weaning compared with Class 1 (*p* < 0.05 for all; Table 2).

Regarding ECMO‐related complications, compared with Class 1, Class 2 was associated with higher risks of bleeding, hyperbilirubinemia, infection, and metabolic complications (*p* < 0.05 for all), while Class 3 was associated with increased risks of renal, hyperbilirubinemia, limb, metabolic, and neurological complications (*p* < 0.05 for all) (Table 2).

### 2.5 Heterogeneity of Treatment Effect for IABP Across Lactate Trajectory Classes

#### 2.5.1 Association Between IABP Use and Primary Outcome

**FIGURE 4:**
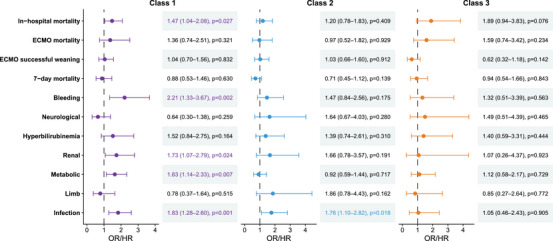
Associations between IABP use and clinical outcomes across different classes. Seven‐day mortality was analyzed using a Cox regression model (HR with 95% CI), and all other outcomes were analyzed using logistic regression models (OR with 95% CI). IABP, intra‐aortic balloon pump; ECMO, extracorporeal membrane oxygenation; OR, odds ratio; HR, hazard ratio; CI, confidence interval.

Multivariable logistic regression analysis showed that in Class 1, IABP use was associated with an increased risk of in‐hospital mortality (OR 1.47, 95% CI 1.04–2.08, *p* = 0.027). In contrast, IABP use was not associated with in‐hospital mortality in Class 2 (OR 1.20, 95% CI 0.78–1.83, *p* = 0.409) or Class 3 (OR 1.89, 95% CI 0.94–3.83, *p* = 0.076) (Figure 4, Table S7). Sensitivity analysis showed that in Class 1, IABP use remained associated with an increased risk of in‐hospital mortality (OR 1.90, 95% CI 1.25–2.89, *p* = 0.002) (Figure S1).

#### 2.5.2 Association Between IABP Use and Secondary Outcomes

Multivariable regression analysis indicated that IABP use was not associated with ECMO mortality, ECMO successful weaning, or 7‐day mortality across the three classes (Figure 4, Table S7). However, in Class 1, IABP use was associated with increased risks of bleeding (OR 2.21, 95% CI 1.33–3.67, *p* = 0.002), renal (OR 1.73, 95% CI 1.07–2.79, *p* = 0.024), metabolic (OR 1.63, 95% CI 1.14–2.33, *p* = 0.007), and infectious (OR 1.83, 95% CI 1.28–2.60, *p* = 0.001) complications. In Class 2, IABP use was associated with an increased risk of infectious complications (OR 1.76, 95% CI 1.10–2.82, *p* = 0.018) (Figure 4, Table S7). The sensitivity analysis in Class 1 showed consistent results (Figure S1).

## 3 Discussion

### 3.1 Main Findings

In this study of AMICS patients receiving VA‐ECMO support, three distinct lactate trajectories classes were identified based on early lactate dynamics before and after ECMO initiation. The results demonstrated significant differences in mortality and ECMO‐related complications among the classes. More importantly, substantial heterogeneity of treatment effect for IABP was observed across the classes. In the “low–slow decline” phenotype, IABP use was associated with increased risks of in‐hospital mortality and multiple ECMO‐related complications, whereas no similar trend was observed among patients with initially elevated lactate levels. These findings highlight that dynamic lactate trajectories not only reflect metabolic recovery status but may also predict responsiveness to mechanical circulatory support, offering new insights for precision management in VA‐ECMO therapy.

### 3.2 Mechanistic Interpretation of Differences Across Lactate Trajectory Classes

The three lactate trajectories reflect distinct pathophysiological states and varying degrees of metabolic reversibility. First, patients with the “low–slow decline” phenotype exhibited relatively mild illness, with only modest initial lactate elevation followed by a gradual decline, suggesting minor tissue hypoperfusion and limited lactate production; the slow metabolic decline may be attributed to mild hepatic or renal dysfunction. Their higher blood pressure and lower intervention intensity indicate preserved circulatory compensation, which explains the more favorable prognosis. In contrast, patients in the “high–rapid decline” phenotype presented with markedly elevated lactate levels at admission, indicating severe oxygen supply–demand imbalance. However, the rapid subsequent decrease reflected effective tissue reperfusion and good metabolic reversibility, suggesting that early ECMO initiation and sustained circulatory support provided clear metabolic benefits. This finding is consistent with the results of the Dobutamine Compared to Milrinone in the Treatment of Cardiogenic Shock (DOREMI) trial, which demonstrated that a rapid and early decline in lactate levels toward normalization was associated with higher survival rates [28]. Finally, the “high–delayed decline” phenotype demonstrated delayed improvement. The sustained early lactate elevation indicated that hypoperfusion was not promptly corrected or that mitochondrial dysfunction was present, while the later decline might have resulted from delayed resuscitation, ongoing inflammation, or a lag in organ function recovery [29]. Therefore, dynamic lactate trajectories may serve as an integrated indicator of tissue perfusion and metabolic recovery rate, providing mechanistic insight into the metabolic recovery processes of different patient subgroups.

### 3.3 Clinical Implications of Heterogeneity of Treatment Effect for IABP

In this multicenter AMICS cohort, we demonstrate for the first time that lactate trajectories not only reflect underlying pathophysiology but also reveal heterogeneity of treatment effect for IABP. Specifically, IABP use was associated with higher in‐hospital mortality in patients with a “low–slow decline” phenotype, whereas its effect was neutral among patients with high baseline lactate levels. This heterogeneity of treatment effect aligns closely with existing evidence. Although IABP was historically considered capable of improving hemodynamics in CS—and some studies from the thrombolytic era even suggested potential survival benefits [30, 31, 32]—consistent findings from the IABP‐SHOCK II trial and its long‐term follow‐up [8, 33], along with multiple large registries and meta‐analyses [9, 34, 35], indicate that among AMICS patients treated with early percutaneous coronary intervention (PCI), IABP confers no survival benefit and instead increases the risks of bleeding, vascular complications, infection, and perfusion instability [36, 37]. Consequently, the ESC guidelines downgraded IABP to a Class III recommendation [13, 14, 15].

Lactate trajectories offer a critical lens for interpreting this differential treatment effect. Patients with a low–slow decline trajectory typically retain relatively preserved tissue perfusion and compensatory capacity, reflecting a more stable clinical course. In such individuals, introducing IABP may disrupt existing compensatory mechanisms and thereby precipitate hemodynamic deterioration. Moreover, the inherent risks of IABP, including bleeding, limb ischemia, and infection, are more likely to translate into adverse outcomes in lower‐risk patients [37]. Additionally, in real‐world practice, IABP use frequently co‐occurs with more aggressive interventional strategies, such as intensified antithrombotic therapy, multivessel PCI, and thrombectomy, which can further amplify its detrimental impact [36]. From a pathophysiological perspective, IABP augments diastolic pressure to enhance coronary perfusion and reduces left ventricular afterload through presystolic deflation [38, 39]. However, in patients with preserved compensatory mechanisms, such modulation of the arterial pressure waveform and afterload may disrupt the existing neurohumoral balance and interfere with compensatory vascular tone regulation. In addition, IABP may influence heart rate and alter left ventricular filling dynamics, thereby affecting the myocardial oxygen supply–demand relationship. If the improvement in coronary perfusion is limited while myocardial oxygen consumption increases, a net detrimental effect may occur. In patients with relatively milder disease severity, the risks associated with IABP‐related complications are more likely to translate into an overall unfavorable clinical impact.

In contrast, patients with high baseline lactate levels are typically already in profound shock, characterized by severe hypoperfusion, metabolic derangement, and multiple organ dysfunction. In this setting, prognosis is primarily driven by the intrinsic severity of shock, and the limited mechanical support provided by IABP is insufficient to alter the disease trajectory, resulting in an overall neutral effect.

### 3.4 Study Limitations

This study has several limitations. First, as a retrospective analysis of registry data, this study is subject to several inherent limitations. Despite multivariable adjustment, risks of selection bias and unmeasured confounding cannot be entirely excluded. In addition, variations in clinical practice across participating centers may have influenced both treatment decisions and outcomes. The observational nature of this study cannot draw causal conclusions. Second, the lactate trajectories were constructed based on a limited number of time points before and after ECMO initiation, without covering the entire treatment course, which may have constrained the precision and stability of the classification. However, the sensitivity analysis supported the reliability of the study findings. Third, patients with fewer than two lactate measurements were excluded. These patients were likely less severely ill and underwent less frequent monitoring (Table S1); therefore, the results may affect the generalizability of findings to other countries. Finally, the timing of IABP insertion relative to VA‐ECMO initiation was not available for most patients in this registry. Although a few cases had timing records, the data were insufficient for reliable analysis or adjustment. Since IABP may be placed before, during, or after VA‐ECMO initiation—each with potentially different physiological effects and risks—the lack of timing information is an important confounding factor. Also, the decision to use IABP was not standardized across centers, which may have affected the interpretation of the findings. Future studies with prospectively collected procedural data are needed to determine whether IABP timing modifies the treatment effects observed in our study.

## 4 Conclusions

Lactate trajectories can stratify mortality risk in AMICS patients receiving VA‐ECMO support and reflect heterogeneous responses to IABP treatment.

## 5 Materials and Methods

### 5.1 Data Source

This multicenter retrospective cohort study was reported in compliance with the Strengthening the Reporting of Observational Studies in Epidemiology (STROBE) statement [40]. Data were obtained from the Chinese Society Extracorporeal Life Support (CSECLS) [10], a nationwide database that collects clinical information on VA‐ECMO use in both adult and pediatric populations across more than 112 centers in China. All participating centers report data through a standardized electronic case form via the official CSECLS web platform. This retrospective study was approved by the Ethics Committee of Beijing Anzhen Hospital, Capital Medical University (Approval No. 2019040X), which also waived the requirement for informed consent. The study was conducted in accordance with the Declaration of Helsinki.

We included hospitalized patients with AMICS who received VA‐ECMO support between January 2017 and February 2025. Exclusion criteria were as follows: age < 18 years; VA‐ECMO support duration < 24 h; hospital stay < 24 h; initial ECMO mode other than VA‐ECMO; and missing or incomplete lactate data (defined as fewer than two measurements among the three required time points: 6 h before, 4 h after, and 24 h after ECMO initiation).

### 5.2 Outcomes and Definitions

The primary outcome was in‐hospital mortality. Secondary outcomes included ECMO mortality (death during ECMO support), ECMO successful weaning (survival 48 h after successful weaning from ECMO), 7‐day mortality (death within seven days of hospital admission), and ECMO‐related complications, including bleeding, neurological events, hyperbilirubinemia, renal, infectious, metabolic, and limb complications; see the Supporting Information for detailed definitions of outcomes.

### 5.3 Data Collection and Management

Variables included demographic information, medical history, pre‐ECMO vital signs, arterial blood gas before and after ECMO initiation, treatment interventions, outcomes, and ECMO‐related complications. Lactate values were collected at three time points: 6 h before, 4 h after, and 24 h after ECMO initiation. Patients with measurements available for at least two of the three time points were included in the analysis. This time window was selected based on the following rationale: (1) clinical relevance: the three time points correspond to distinct physiological phases during ECMO support, enabling dynamic assessment before and after circulatory support; (2) established validity in prior studies: lactate within the first 24 h of ECMO support has been consistently demonstrated to be a robust predictor of mortality in patients with refractory CS [19]; and (3) methodological requirements for trajectory modeling: LCGM requires at least three repeated measurements to reliably identify distinct trajectory patterns while preserving trajectory validity [41].

For vital signs and arterial blood gas variables, outliers were identified using the percentile method, with values below the first percentile or above the 99th percentile considered abnormal and set as missing; the proportion of outliers for each variable is shown in Figure S2. Variables with more than 40% missing values were excluded (Figure S3). For variables included in the analysis, multiple imputation was performed using five imputation algorithms (mean, predictive mean matching, Lasso. Norm, random forest, and classification and regression tree) to generate five imputed datasets. The mean of the imputed values across datasets was used for the final analysis [42]. Missing lactate values at specific time points were not imputed but were handled within the LCGM framework using likelihood‐based estimation.

### 5.4 Development of the Lactate Trajectory Classes

The lactate trajectory modeling process consisted of the following steps: first, an LCGM was applied to determine the optimal number of latent classes. Based on previous studies [22, 23], models with two to six classes were considered. The optimal number of classes was determined by BIC and clinical interpretability. After determining the optimal number of classes, a growth mixture modeling (GMM) was used to fit the trajectories, considering linear, quadratic, and cubic polynomial forms, as well as two random effects structures (random intercept and random slope + intercept). In total, six GMM models were constructed to identify the best‐fitting trajectory model. Subsequently, each patient was assigned to the trajectory group corresponding to the maximum posterior probability, with an average posterior probability > 0.7 used as the criterion for classification robustness. To avoid model overfitting and maintain parsimony, each latent class was required to include at least 10% of the total study population.

### 5.5 Statistical Analysis

All statistical analyses were performed using R version 4.5.0. Continuous variables were expressed as mean ± standard deviation for normally distributed data, or median (interquartile range) for non‐normally distributed data. Categorical variables were presented as counts (percentages). Group comparisons were conducted using one‐way analysis of variance or the Kruskal–Wallis test for continuous variables, and chi‐square test or Fisher's exact test for categorical variables.

Logistic regression models were used to evaluate associations between lactate trajectory classes and in‐hospital mortality, ECMO mortality, ECMO successful weaning, and ECMO‐related complications. Cox proportional hazards models were applied to assess associations between lactate trajectory classes and 7‐day mortality, and adjusted Kaplan–Meier curves and log‐rank tests comparing survival across classes. Three progressively adjusted models were developed: Model 1 (unadjusted), Model 2 (adjusted for age and sex), and Model 3 (further adjusted for body mass index, medical history, pre‐ECMO cardiac arrest, pre‐ECMO vasopressors, and pre‐ECMO mechanical ventilation). To assess multicollinearity among the covariates included in Model 3, we calculated variance inflation factors (VIFs). All VIF values were below 5, indicating no substantial multicollinearity. Detailed VIF results are provided in Figure S4. Subgroup analyses were performed to evaluate associations between trajectory classes and in‐hospital mortality across different strata, including age, sex, IABP use, hypertension, diabetes mellitus, myocardial infarction, heart failure, and pre‐ECMO cardiac arrest. The class with the largest sample size served as the reference group.

To explore the heterogeneity of treatment effect for IABP use across trajectory classes, patients were divided into IABP and non‐IABP groups based on whether IABP was used. Multivariable analysis was applied to assess the association between IABP use and outcomes within each class, adjusting for the same covariates as in Model 3.

Additionally, a sensitivity analysis was conducted, including only patients with complete lactate measurements at all three time points for trajectory modeling, in order to reassess heterogeneity of treatment effect for IABP use. A two‐sided *p* < 0.05 was considered statistically significant.

## Author Contributions

**Wan‐Jie Gu**: conceptualization, supervision, writing – review and editing, funding acquisition. **Hai‐Yan Yin**: methodology, funding acquisition, project administration. **Xiao‐Tong Hou**: data curation, resources, project administration. **Xiang‐Jie Duan**: conceptualization, methodology, software, visualization, writing – original draft. **Bo Wang**: investigation, formal analysis. **Cheng‐Long Li**: data curation, resources. **Wan Chen**: investigation. **Peng Ding**: visualization, writing – review and editing. **Jie‐Lian Zhu**: Validation. **Jun‐Wei Wang**: Validation. All authors have read and approved the final manuscript.

## Funding

This work was supported by the National Natural Science Foundation of China (No. 82572459 to WJG), the Science and Technology Projects of Guangzhou (No. 2025A03J4248, 2025A03J3472, and 2025A04J3478 to HYY), the Project of the Guangdong–Hong Kong–Macao Greater Bay Area as an International Innovation and Technology Hub (No. 2025A0505010023 to HYY), and the State Key Laboratory of Neurology and Oncology Drug Development (No. SKLSIM‐F‐2025063 to HYY and SKLSIM‐F‐2025096 to WJG).

## Ethics Statement

The study protocol was approved by the Ethics Committee of Beijing Anzhen Hospital, Capital Medical University (2019040X), and conducted in accordance with the Declaration of Helsinki.

## Conflicts of Interest

The authors declare no conflicts of interest.

## Supporting information




**Supporting Table 1**: Clinical characteristics of included and excluded AMICS patients.
**Supporting Table 2**: Comparison of LCGM models for determining the number of latent classes.
**Supporting Table 3**: Comparison of GMM models for determining the optimal trajectory.
**Supporting Table 4**: Mean of posterior probabilities in each lactate class.
**Supporting Table 5**: Fixed effects in the longitudinal three classes model.
**Supporting Table 6**: Association between different lactate trajectories and in‐hospital mortality across subgroups.
**Supporting Table 7**: Association between IABP use and outcomes across different classes.
**Supporting Figure 1**: Associations between IABP use and clinical outcomes across different classes in sensitivity analysis.
**Supporting Figure 2**: Percentages of outlier data for all continuous variables included in study population.
**Supporting Figure 3**: Percentages of missing data for all included variables in study population.
**Supporting Figure 4**: Variance inflation factors for variables included in model 3 for each outcome. A variance inflation factor < 5 for each variable suggested the absence of multicollinearity. Abbreviations: BMI, body mass index; ECMO, extracorporeal membrane oxygenation.

## Data Availability

Detailed data and statistical analysis codes are available from the corresponding author upon request.
