# Supplementary material for: Heterogeneity of Treatment Effect for Intra‐Aortic Balloon Pump Use Across Lactate Trajectories in Cardiogenic Shock Patients Supported by VA‐ECMO: An Analysis of the Chinese Extracorporeal Life Support Registry
Source: MedComm (2020). 2026 Mar 18;7(4):e70698. doi: 10.1002/mco2.70698 (PMC13042427; doi:10.1002/mco2.70698)
Supplement: Supplementary file 1 — Supporting Table 1: Clinical characteristics of included and excluded AMICS patients. Supporting Table 2: Comparison of LCGM models for determining the number of latent classes. Supporting Table 3: Comparison of GMM models for determining the optimal trajectory. Supporting Table 4: Mean of posterior probabilities in each lactate class. Supporting Table 5: Fixed effects in the longitudinal three classes model. Supporting Table 6: Association between different lactate trajectories and in‐hospital mortality across subgroups. Supporting Table 7: Association between IABP use and outcomes across different classes. Supporting Figure 1: Associations between IABP use and clinical outcomes across different classes in sensitivity analysis. Supporting Figure 2: Percentages of outlier data for all continuous variables included in study population. Supporting Figure 3: Percentages of missing data for all included variables in study population. Supporting Figure 4: Variance inflation factors for variables included in model 3 for each outcome. A variance inflation factor < 5 for each variable suggested the absence of multicollinearity. Abbreviations: BMI, body mass index; ECMO, extracorporeal membrane oxygenation. [file MCO2-7-e70698-s001.docx]

**Heterogeneity of treatment effect for intra-aortic balloon pump use across lactate trajectories in cardiogenic shock patients supported by VA-ECMO: an analysis of the Chinese Extracorporeal Life Support Registry**

Xiang-Jie Duan, MD, PhD^1,#^, Bo Wang, MD, Msc^2,3#^, Cheng-Long Li, MD, PhD^4,#^, Wan Chen, MD, Msc^3^, Peng Ding, MD, PhD^1^, Jie-Lian Zhu, MD, Msc^1^, Jun-Wei Wang, MD, Msc^1^, Xiao-Tong Hou, MD, PhD^4,#^, Hai-Yan Yin, MD, PhD^1,*^, Wan-Jie Gu, MD, PhD^1,*^

**Table of contents**

| Item | Title |
| --- | --- |
| Materials and Methods | Outcomes and definitions |
| Table S1 | Clinical characteristics of included and excluded AMICS patients. |
| Table S2 | Comparison of LCGM models for determining the number of latent classes. |
| Table S3 | Comparison of GMM models for determining the optimal trajectory. |
| Table S4 | Mean of posterior probabilities in each lactate class. |
| Table S5 | Fixed effects in the longitudinal three classes model. |
| Table S6 | Association between different lactate trajectories and in-hospital mortality across subgroups. |
| Table S7 | Association between IABP use and outcomes across different classes. |
| Figure S1 | Associations between IABP use and clinical outcomes across different classes in sensitivity analysis. |
| Figure S2 | Percentages of outlier data for continuous variables included in study population. |
| Figure S3 | Percentages of missing data for all included variables in study population. |
| Figure S4 | Variance inflation factors for variables included in model 3 for each outcome. |

**Materials and Methods**

**Outcomes and definitions**

**Bleeding complications** included gastrointestinal bleeding, bleeding at the intubation site, surgery-related bleeding, hemolysis, plasma free hemoglobin > 50 mg/dL, and disseminated intravascular coagulation.

**Neurological complications** included cerebral hemorrhage, cerebral infarction, seizures, and brain death.

**Hyperbilirubinemia** was defined as direct bilirubin > 34.48μmol/L, or indirect bilirubin > 224.14μmol/L, or total bilirubin > 258.62μmol/L.

**Renal complications** included serum creatinine > 132.74μmol/L or the need for continuous renal replacement therapy.

**Infectious complications** were defined as culture-proven infection or white blood cell count < 1500 or > 20000/μL.

**Metabolic disturbances** included glucose > 13.3 mmol/L or < 2.2 mmol/L, or pH < 7.2 or > 7.6.

**Limb complications** included distal ischemia, necrosis, fasciotomy, amputation, thrombosis, endoluminal stripping, and nerve injury.

**Table S1. Clinical characteristics of included and excluded AMICS patients.**

| **Characteristics** | **Overall**  **(N=1713)** | **Included**  **(N=1264)** | **Excluded**  **(N=449)** | ***P* value** |
| --- | --- | --- | --- | --- |
| Age (yr) | 60.05 (12.05) | 59.98 (12.07) | 60.26 (11.98) | 0.675 |
| Sex, n (%) |  |  |  | 0.242 |
| Female | 1390 (81.1%) | 1034 (81.8%) | 356 (79.3%) |  |
| Male | 323 (18.9%) | 230 (18.2%) | 93 (20.7%) |  |
| BMI (kg/m^2^) | 24.21 (3.00) | 24.13 (2.86) | 24.44 (3.35) | 0.087 |
| Medical history, n (%) |  |  |  |  |
| Cardiac surgery | 31 (1.8%) | 27 (2.1%) | 4 (0.9%) | 0.089 |
| Cardiac intervention | 332 (19.4%) | 262 (20.7%) | 70 (15.6%) | 0.018 |
| Myocardial infarction | 311 (18.2%) | 246 (19.5%) | 65 (14.5%) | 0.019 |
| Hypertension | 803 (46.9%) | 655 (51.8%) | 148 (33.0%) | <0.001 |
| Diabetes mellitus | 499 (29.1%) | 405 (32.0%) | 94 (20.9%) | <0.001 |
| Hyperlipidemia | 241 (14.1%) | 198 (15.7%) | 43 (9.6%) | 0.001 |
| Heart failure | 224 (13.1%) | 178 (14.1%) | 46 (10.2%) | 0.038 |
| Chronic respiratory diseases | 56 (3.3%) | 46 (3.6%) | 10 (2.2%) | 0.148 |
| Chronic kidney disease | 73 (4.3%) | 57 (4.5%) | 16 (3.6%) | 0.394 |
| Neurological disease | 139 (8.1%) | 116 (9.2%) | 23 (5.1%) | 0.007 |
| Anticoagulants | 158 (9.2%) | 137 (10.8%) | 21 (4.7%) | <0.001 |
| Smoking | 668 (39.0%) | 561 (44.4%) | 107 (23.8%) | <0.001 |
| Pre-ECMO cardiac arrest, n (%) | 501 (29.2%) | 349 (27.6%) | 152 (33.9%) | 0.012 |
| IABP use, n (%) | 739 (43.1%) | 564 (44.6%) | 175 (39.0%) | 0.038 |
| ECMO initiation time (hours) | 1.00 (-1.92, 19.17) | 1.39 (-1.82, 20.68) | 0.03 (-2.00, 15.50) | 0.055 |
| Pre-ECMO support, n (%) |  |  |  |  |
| Mechanical ventilation | 1112 (64.9%) | 953 (75.4%) | 159 (35.4%) | <0.001 |
| Vasopressors use | 1053 (61.5%) | 970 (76.7%) | 83 (18.5%) | <0.001 |
| One type | 459 (26.8%) | 419 (33.1%) | 40 (8.9%) |  |
| Two types | 410 (23.9%) | 377 (29.8%) | 33 (7.3%) |  |
| Three types | 184 (10.7%) | 174 (13.8%) | 10 (2.2%) |  |
| Vasoactive-inotropic score | 18.30 (0.00, 85.90) | 38.00 (4.00, 103.00) | 0.00 (0.00, 0.00) | <0.001 |
| Pre-ECMO hemodynamics |  |  |  |  |
| Heart rate (beats/min) | 107.00 (60.00, 132.00) | 109.00 (62.00, 133.00) | 96.00 (42.00, 130.00) | 0.088 |
| SBP (mmHg) | 74.00 (60.00, 86.00) | 75.00 (60.00, 86.00) | 70.00 (54.00, 82.00) | 0.031 |
| DBP (mmHg) | 45.00 (33.00, 55.00) | 45.00 (34.00, 55.00) | 40.00 (30.00, 53.00) | 0.028 |
| MAP (mmHg) | 54.70 (43.30, 65.30) | 55.70 (43.85, 65.70) | 50.15 (39.30, 63.70) | 0.021 |
| Pre-ECMO blood gases |  |  |  |  |
| pH | 7.26 (0.18) | 7.26 (0.18) | 7.31 (0.16) | 0.029 |
| HCO_3_^-^ (mmol/L) | 17.70 (13.30, 21.10) | 17.60 (13.20, 21.00) | 18.50 (15.10, 22.00) | 0.106 |
| PaO_2_ (mmHg) | 76.00 (59.00, 113.00) | 76.00 (59.00, 113.00) | 79.30 (62.50, 101.00) | 0.782 |
| PaCO_2_ (mmHg) | 36.00 (28.00, 45.00) | 35.95 (28.00, 45.00) | 36.60 (29.00, 43.80) | 0.734 |
| Lactate (mmol/L) | 7.34 (5.35) | 7.34 (5.04) | 7.23 (11.02) | 0.953 |
| SpO_2_ (%) | 88.44 (14.75) | 88.49 (14.84) | 87.35 (12.28) | 0.609 |
| Blood gases for 4 h during ECMO |  |  |  |  |
| pH | 7.37 (0.11) | 7.37 (0.11) | 7.34 (0.14) | 0.386 |
| HCO_3_^-^ (mmol/L) | 20.45 (17.70, 23.00) | 20.50 (17.70, 23.00) | 20.00 (17.50, 23.10) | 0.689 |
| PaO_2_ (mmHg) | 140.00 (89.00, 285.00) | 140.00 (89.00, 286.00) | 135.00 (96.00, 218.00) | 0.802 |
| PaCO_2_ (mmHg) | 35.00 (29.00, 41.00) | 35.00 (29.00, 41.00) | 35.00 (30.90, 42.00) | 0.643 |
| Lactate (mmol/L) | 3.80 (2.10, 7.55) | 3.80 (2.10, 7.50) | 2.65 (1.50, 8.30) | 0.195 |
| SpO_2_ (%) | 97.53 (21.59) | 97.52 (21.82) | 97.95 (2.08) | 0.588 |
| Blood gases for 24 h during ECMO |  |  |  |  |
| pH | 7.42 (0.09) | 7.42 (0.09) | 7.38 (0.11) | 0.060 |
| HCO_3_^-^ (mmol/L) | 23.60 (21.00, 26.30) | 23.70 (21.00, 26.20) | 22.25 (19.70, 26.50) | 0.267 |
| PaO_2_ (mmHg) | 120.00 (89.00, 185.70) | 120.00 (89.20, 184.00) | 111.00 (83.00, 261.00) | 0.953 |
| PaCO_2_ (mmHg) | 36.00 (32.00, 41.90) | 36.00 (32.00, 41.70) | 35.70 (30.00, 43.00) | 0.798 |
| Lactate (mmol/L) | 2.20 (1.40, 3.70) | 2.20 (1.40, 3.60) | 2.33 (1.70, 6.94) | 0.159 |
| SpO_2_ (%) | 97.47 (9.54) | 97.52 (9.57) | 95.02 (7.71) | 0.158 |
| During ECMO support, n (%) |  |  |  |  |
| Mechanical ventilation | 1165 (68.0%) | 1001 (79.2%) | 164 (36.5%) | <0.001 |
| Vasopressors use | 1150 (67.1%) | 1041 (82.4%) | 109 (24.3%) | <0.001 |
| One type | 532 (31.1%) | 481 (38.1%) | 51 (11.4%) |  |
| Two types | 467 (27.3%) | 418 (33.1%) | 49 (10.9%) |  |
| Three types | 151 (8.8%) | 142 (11.2%) | 9 (2.0%) |  |
| Vasoactive-inotropic score | 10.00 (0.00, 50.00) | 20.00 (4.00, 60.00) | 0.00 (0.00, 0.00) | <0.001 |
| ECMO assistance (hour) | 116.50 (67.83, 184.67) | 118.74 (71.00, 189.50) | 111.00 (58.67, 173.00) | 0.005 |
| Length of hospital stay (days) | 13.62 (6.88, 22.41) | 14.01 (7.90, 22.67) | 12.38 (5.07, 22.00) | 0.002 |
| Length of ICU stay (days) | 10.52 (5.52, 16.83) | 10.65 (5.82, 16.94) | 9.55 (3.86, 15.91) | 0.026 |
| In-hospital mortality, n (%) | 687 (40.1%) | 524 (41.5%) | 163 (36.3%) | 0.056 |
| ECMO mortality, n (%) | 185 (10.8%) | 146 (11.6%) | 39 (8.7%) | 0.093 |
| ECMO successful weaning, n (%) | 1212 (70.8%) | 899 (71.1%) | 313 (69.7%) | 0.572 |
| 7-day mortality, n (%) | 300 (17.5%) | 211 (16.7%) | 89 (19.8%) | 0.134 |
| Complications, n (%) |  |  |  |  |
| Bleeding | 217 (12.7%) | 180 (14.2%) | 37 (8.2%) | 0.001 |
| Neurological | 100 (5.8%) | 79 (6.3%) | 21 (4.7%) | 0.222 |
| Renal | 1521 (88.8%) | 1109 (87.7%) | 412 (91.8%) | 0.020 |
| Metabolic | 554 (32.3%) | 416 (32.9%) | 138 (30.7%) | 0.397 |
| Limb | 99 (5.8%) | 80 (6.3%) | 19 (4.2%) | 0.102 |
| Hyperbilirubinemia | 175 (10.2%) | 142 (11.2%) | 33 (7.3%) | 0.020 |
| Infection | 434 (25.3%) | 367 (29.0%) | 67 (14.9%) | <0.001 |

Continuous data are expressed as mean (SD) or median (IQR). ECMO initiation time defined by the interval between hospital admission and ECMO initiation.

Abbreviations: BMI, body mass index; ECMO, extracorporeal membrane oxygenation; IABP, intra-aortic balloon pump; SBP, systolic blood pressure; DBP, diastolic blood pressure; MAP, mean arterial pressure; ICU, intensive care unit; AMICS, acute myocardial infarction-related cardiogenic shock. SD, standard deviation; IQR, interquartile range.

**Table S2. Comparison of LCGM models for determining the number of latent classes.**

| **LCGM** | **Number of Classes** | **Loglik** | **AIC** | **BIC** | **SABIC** | **Entropy** | **ICL** | **% Class 1** | **% Class 2** | **% Class 3** | **% Class 4** | **% Class 5** | **% Class 6** |
| --- | --- | --- | --- | --- | --- | --- | --- | --- | --- | --- | --- | --- | --- |
| M2 | 2 | -9318.64 | 18649.29 | 18680.14 | 18661.08 | 0.895 | 18771.85 | 81.65 | 18.35 |  |  |  |  |
| M3 | 3 | -9097.83 | 18213.66 | 18259.94 | 18231.35 | 0.907 | 18389.48 | 4.11 | 76.82 | 19.07 |  |  |  |
| M4 | 4 | -9025.22 | 18074.45 | 18136.15 | 18098.04 | 0.837 | 18421.70 | 3.80 | 68.20 | 19.94 | 8.07 |  |  |
| M5 | 5 | -9025.22 | 18080.45 | 18157.58 | 18109.93 | 0.608 | 18954.30 | 67.09 | 21.04 | 3.80 | 0.00 | 8.07 |  |
| M6 | 6 | -9025.22 | 18086.45 | 18179.01 | 18121.83 | 0.506 | 19298.48 | 0.00 | 8.07 | 3.80 | 65.27 | 22.86 | 0.00 |

Abbreviations: LCGM: latent class growth modeling; Loglik: log-likelihood; AIC: akaike information criterion; BIC: bayesian information criterion; SABIC: sample-size adjusted BIC; ICL: integrated completed likelihood criterion.

Loglik represents the log-likelihood value, where a larger value (closer to 0) indicates a better model fit. AIC, BIC, SABIC, and ICL are model selection criteria, with smaller values reflecting a better model fit. Entropy measures the precision of class classification, where values closer to 1 indicate greater clarity and distinctiveness among classes. “% Class 2–6” represents the proportion of samples in each class.

Among all constructed models, the M4 model yielded the lowest AIC, BIC, and SABIC values; however, the reductions compared with the M3 model were minimal. In addition, the entropy value decreased (from 0.907 to 0.837), and the ICL value increased, suggesting a potential risk of overfitting. Furthermore, compared with the M3 model, the M4 model did not show improved differentiation in class proportions. Therefore, in this study, K = 3 was determined to be the optimal number of latent classes.

**Table S3. Comparison of GMM models for determining the optimal trajectory.**

| **GMM** | **Number of Classes** | **Loglik** | **AIC** | **BIC** | **SABIC** | **Entropy** | **ICL** | **% Class 1** | **% Class 2** | **% Class 3** |
| --- | --- | --- | --- | --- | --- | --- | --- | --- | --- | --- |
| M1 | 3 | -8971.89 | 17967.77 | 18029.48 | 17991.36 | 0.831 | 18263.59 | 72.31 | 5.38 | 22.31 |
| M2 | 3 | -8903.38 | 17834.76 | 17906.75 | 17862.28 | 0.745 | 18260.24 | 66.06 | 5.22 | 28.72 |
| M3 | 3 | -8886.38 | 17802.76 | 17879.89 | 17832.24 | 0.814 | 18138.11 | 69.22 | 8.07 | 22.71 |
| M4 | 3 | -8698.13 | 17430.27 | 17517.68 | 17463.68 | 0.753 | 17860.96 | 55.93 | 29.91 | 14.16 |
| M5 | 3 | -9487.26 | 19010.52 | 19103.08 | 19045.90 | 1.000 | 19103.08 | 0.00 | 100.00 | 0.00 |
| M6 | 3 | -9384.80 | 18809.59 | 18912.43 | 18848.90 | 1.000 | 18912.43 | 0.00 | 100.00 | 0.00 |

Abbreviations: GMM: growth mixture modeling; Loglik: log-likelihood; AIC: akaike information criterion; BIC: bayesian information criterion; SABIC: sample-size adjusted BIC; ICL: integrated completed likelihood criterion.

The results indicated that among the six GMM models incorporating linear, quadratic, and cubic polynomials with random intercepts and random slopes, the M4 model yielded the lowest AIC, BIC, SABIC, and ICL values, with entropy exceeding 0.7, indicating good model discrimination. Furthermore, the M4 model exhibited the most balanced sample distribution, with each of the three latent subphenotypes accounting for at least 10% of the total population. Therefore, the M4 model was identified as the optimal trajectory model for this study.

**Table S4. Mean of posterior probabilities in each lactate class.**

| **Class** | **Probability 1** | **Probability 2** | **Probability 3** |
| --- | --- | --- | --- |
| 1 | 0.9037 | 0.0890 | 0.0073 |
| 2 | 0.0807 | 0.8662 | 0.0531 |
| 3 | 0.0207 | 0.0654 | 0.9139 |

The mean posterior probabilities of group membership for the group members were all above 70%.

**Table S5. Fixed effects in the longitudinal three classes model.**

| **Items** | **Coefficient** | **Standard error** | **Wald statistic** | ***P* value** |
| --- | --- | --- | --- | --- |
| intercept class1 | 3.16115 | 0.09553 | 33.091 | <0.001 |
| intercept class2 | 7.94223 | 0.24664 | 32.202 | <0.001 |
| intercept class3 | 13.07622 | 0.45682 | 28.624 | <0.001 |
| poly1 class1 | -0.09802 | 0.01201 | -8.164 | <0.001 |
| poly1 class2 | -0.47177 | 0.02051 | -22.998 | <0.001 |
| poly1 class3 | 0.38757 | 0.03615 | 10.722 | <0.001 |
| Poly2 class1 | 0.00198 | 0.00051 | 3.892 | <0.001 |
| Poly2 class2 | 0.01232 | 0.00084 | 14.609 | <0.001 |
| Poly2 class3 | -0.02648 | 0.00131 | -20.170 | <0.001 |

All parameters were statistically significant (*P* < 0.001), indicating clear distinctions among the trajectories. Class 1 demonstrated the lowest baseline level with a slight downward trend, Class 2 exhibited a higher initial level followed by a marked decline that gradually plateaued, and Class 3 showed the highest baseline level with an upward trend that later stabilized. These findings confirm substantial heterogeneity in baseline values and temporal dynamics across the three latent classes.

**Table S6. Association between different lactate trajectories and in-hospital mortality across subgroups.**

| **Variables** | **Cases/percent** | **Class 1** | **Class 2** | **Class 3** | ***P* for interaction** |
| --- | --- | --- | --- | --- | --- |
| Total | 1264 (100.0%) | Reference | 2.03 (1.54, 2.67) | 3.99 (2.76, 5.75) |  |
| Age, (yr) |  |  |  |  | 0.064 |
| < 65 | 799 (63.2%) | Reference | 2.63 (1.84, 3.76) | 5.74 (3.48, 9.47) |  |
| ≥ 65 | 465 (36.8%) | Reference | 1.37 (0.88, 2.14) | 2.68 (1.53, 4.72) |  |
| Sex |  |  |  |  | 0.428 |
| Male | 1034 (81.8%) | Reference | 2.01 (1.48, 2.72) | 4.57 (3.00, 6.96) |  |
| Female | 230 (18.2%) | Reference | 2.77 (1.37, 5.59) | 3.62 (1.56, 8.39) |  |
| IABP use |  |  |  |  | 0.580 |
| No | 700 (55.4%) | Reference | 2.17 (1.49, 3.17) | 3.69 (2.20, 6.21) |  |
| Yes | 564 (44.6%) | Reference | 1.90 (1.26, 2.88) | 4.65 (2.66, 8.12) |  |
| Hypertension | | | | | 0.055 |
| No | 609 (48.2%) | Reference | 2.91 (1.93, 4.38) | 5.51 (3.15, 9.65) |  |
| Yes | 655 (51.8%) | Reference | 1.58 (1.08, 2.31) | 3.04 (1.84, 5.01) |  |
| Diabetes mellitus | | | | | 0.933 |
| No | 859 (68.0%) | Reference | 2.17 (1.54, 3.05) | 4.13 (2.61, 6.53) |  |
| Yes | 405 (32.0%) | Reference | 2.05 (1.26, 3.34) | 3.85 (2.05, 7.22) |  |
| Myocardial infarction | | | | | 0.206 |
| No | 1018 (80.5%) | Reference | 1.83 (1.36, 2.48) | 3.90 (2.54, 5.98) |  |
| Yes | 246 (19.5%) | Reference | 4.19 (2.01, 8.71) | 5.67 (2.58, 12.43) |  |
| Heart failure | | | | | 0.879 |
| No | 1086 (85.9%) | Reference | 1.98 (1.48, 2.66) | 3.88 (2.59, 5.81) |  |
| Yes | 178 (14.1%) | Reference | 2.34 (0.99, 5.54) | 5.16 (1.93, 13.78) |  |
| Pre-ECMO cardiac arrest | | | | | 0.665 |
| No | 915 (72.4%) | Reference | 1.92 (1.38, 2.66) | 3.65 (2.35, 5.68) |  |
| Yes | 349 (27.6%) | Reference | 2.66 (1.56, 4.53) | 6.21 (3.01, 12.83) |  |

Odds ratios (ORs) with 95% confidence intervals (CIs) was calculated using a logistic regression model. The model was adjusted for age, sex, BMI, smoking, cardiac surgery, cardiac intervention, anticoagulants, hypertension, diabetes mellitus, heart failure, myocardial infarction, hyperlipidemia, chronic respiratory diseases, neurological disease, chronic kidney disease, pre-ECMO cardiac arrest, pre-ECMO mechanical ventilation, and pre-ECMO vasopressors.

Abbreviations: BMI, body mass index; ECMO, extracorporeal membrane oxygenation; IABP, intra-aortic balloon pump.

**Table S7. Association between IABP use and outcomes across different classes.**

| **Outcomes** | **Class 1** | | **Class 2** | | **Class 3** | | ***P* for interaction** |
| --- | --- | --- | --- | --- | --- | --- | --- |
|  | **OR/HR (95% CI)** | ***P* value** | **OR/HR (95% CI)** | ***P* value** | **OR/HR (95% CI)** | ***P* value** |  |
| In-hospital mortality | 1.47 (1.04, 2.08) | 0.027 | 1.20 (0.78, 1.83) | 0.409 | 1.89 (0.94, 3.83) | 0.076 | 0.580 |
| ECMO mortality | 1.36 (0.74, 2.51) | 0.321 | 0.97 (0.52, 1.82) | 0.929 | 1.59 (0.74, 3.42) | 0.234 | 0.536 |
| ECMO successful weaning | 1.04 (0.70, 1.56) | 0.832 | 1.03 (0.66, 1.61) | 0.912 | 0.62 (0.32, 1.18) | 0.142 | 0.292 |
| 7-day mortality | 0.88 (0.53, 1.46) | 0.630 | 0.71 (0.45, 1.12) | 0.139 | 0.94 (0.54, 1.66) | 0.843 | 0.812 |
| Complications |  |  |  |  |  |  |  |
| Bleeding | 2.21 (1.33, 3.67) | 0.002 | 1.47 (0.84, 2.56) | 0.175 | 1.32 (0.51, 3.39) | 0.563 | 0.322 |
| Neurological | 0.64 (0.30, 1.38) | 0.259 | 1.64 (0.67, 4.03) | 0.280 | 1.49 (0.51, 4.39) | 0.465 | 0.116 |
| Metabolic | 1.63 (1.14, 2.33) | 0.007 | 0.92 (0.59, 1.44) | 0.717 | 1.12 (0.58, 2.17) | 0.729 | 0.274 |
| Limb | 0.78 (0.37, 1.64) | 0.515 | 1.86 (0.78, 4.43) | 0.162 | 0.85 (0.27, 2.64) | 0.772 | 0.604 |
| Hyperbilirubinemia | 1.52 (0.84, 2.75) | 0.164 | 1.39 (0.74, 2.61) | 0.310 | 1.40 (0.59, 3.31) | 0.444 | 0.767 |
| Renal | 1.73 (1.07, 2.79) | 0.024 | 1.66 (0.78, 3.57) | 0.191 | 1.07 (0.26, 4.37) | 0.923 | 0.871 |
| Infection | 1.83 (1.28, 2.60) | 0.001 | 1.76 (1.10, 2.82) | 0.018 | 1.05 (0.46, 2.43) | 0.905 | 0.693 |

The model was adjusted for age, sex, BMI, smoking, cardiac surgery, cardiac intervention, anticoagulants, hypertension, diabetes mellitus, heart failure, myocardial infarction, hyperlipidemia, chronic respiratory diseases, neurological disease, chronic kidney disease, pre-ECMO cardiac arrest, pre-ECMO mechanical ventilation, and pre-ECMO vasopressors.

7-day mortality was analyzed using a Cox regression model (HR with 95% CI), and all other outcomes were analyzed using logistic regression models (OR with 95% CI).

Abbreviations: BMI, body mass index; ECMO, extracorporeal membrane oxygenation; OR, Odds Ratio; HR, Hazard Ratio; CI, Confidence Interval.

**
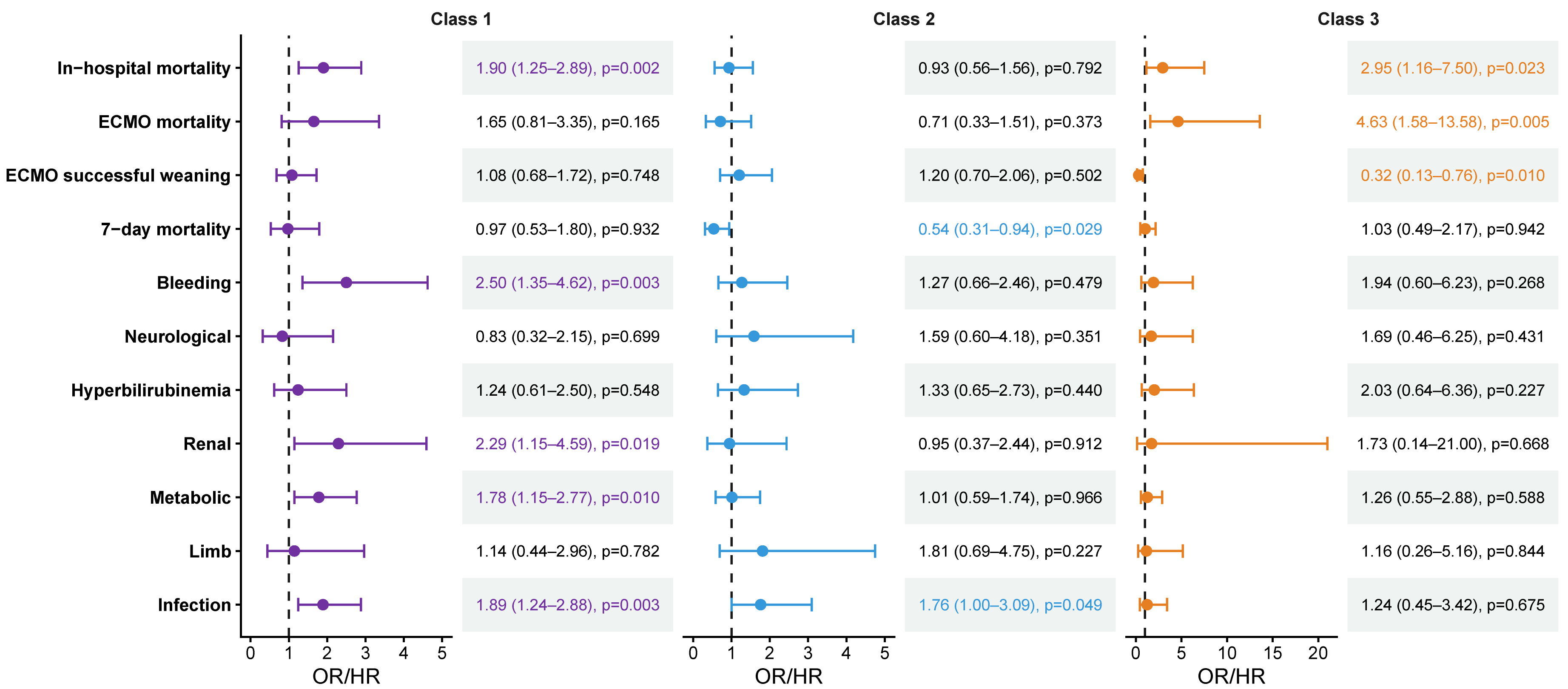
**

**Figure S1. Associations between IABP use and clinical outcomes across different classes in sensitivity analysis.** The model was adjusted for age, sex, BMI, smoking, cardiac surgery, cardiac intervention, anticoagulants, hypertension, diabetes mellitus, heart failure, myocardial infarction, hyperlipidemia, chronic respiratory diseases, neurological disease, chronic kidney disease, pre-ECMO cardiac arrest, pre-ECMO mechanical ventilation, and pre-ECMO vasopressors.

7-day mortality was analyzed using a Cox regression model (HR with 95% CI), and all other outcomes were analyzed using logistic regression models (OR with 95% CI).

Abbreviations: BMI, body mass index; ECMO, extracorporeal membrane oxygenation; IABP: intra-aortic balloon pump; OR, odds ratio; HR, hazard ratio; CI, Confidence Interval.


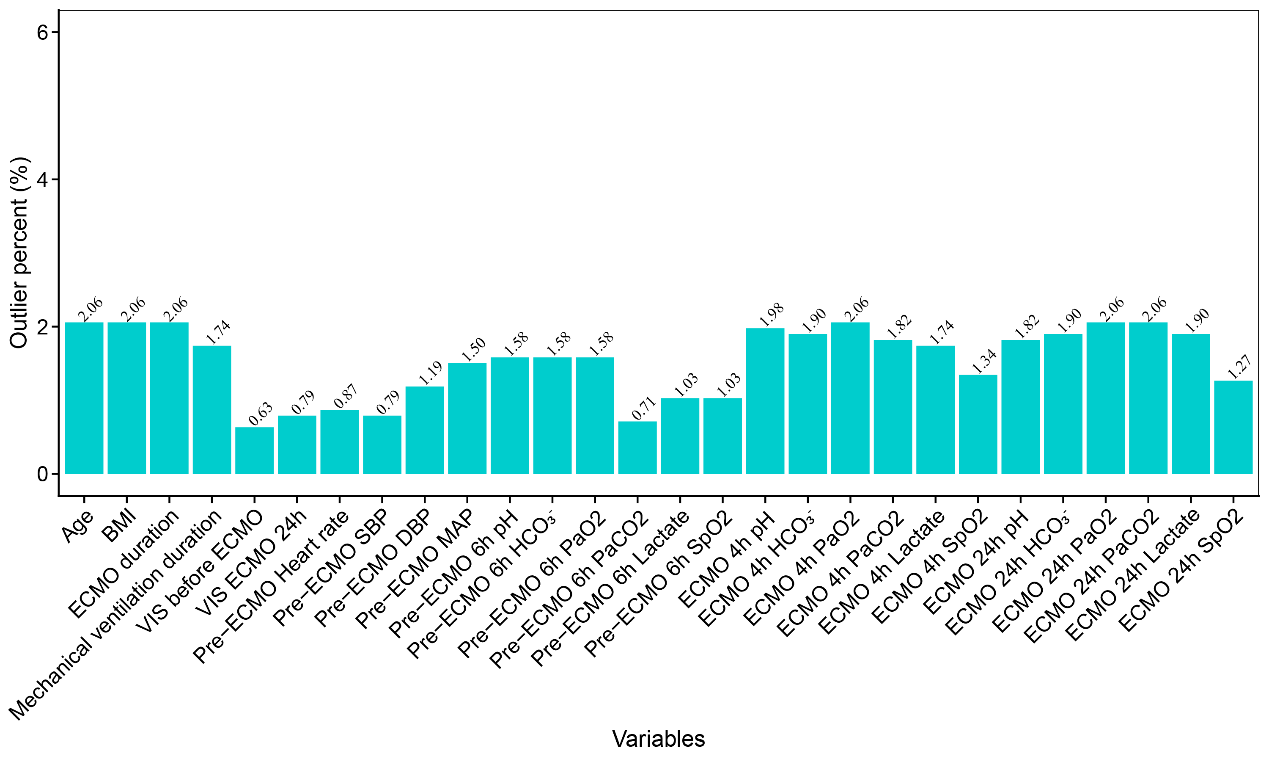


**Figure S2. Percentages of outlier data for all continuous variables included in study population.**

Abbreviations: BMI, body mass index; ECMO, extracorporeal membrane oxygenation; VIS, vasoactive-inotropic score; SBP, systolic blood pressure; DBP, diastolic blood pressure; MAP, mean arterial pressure.

**
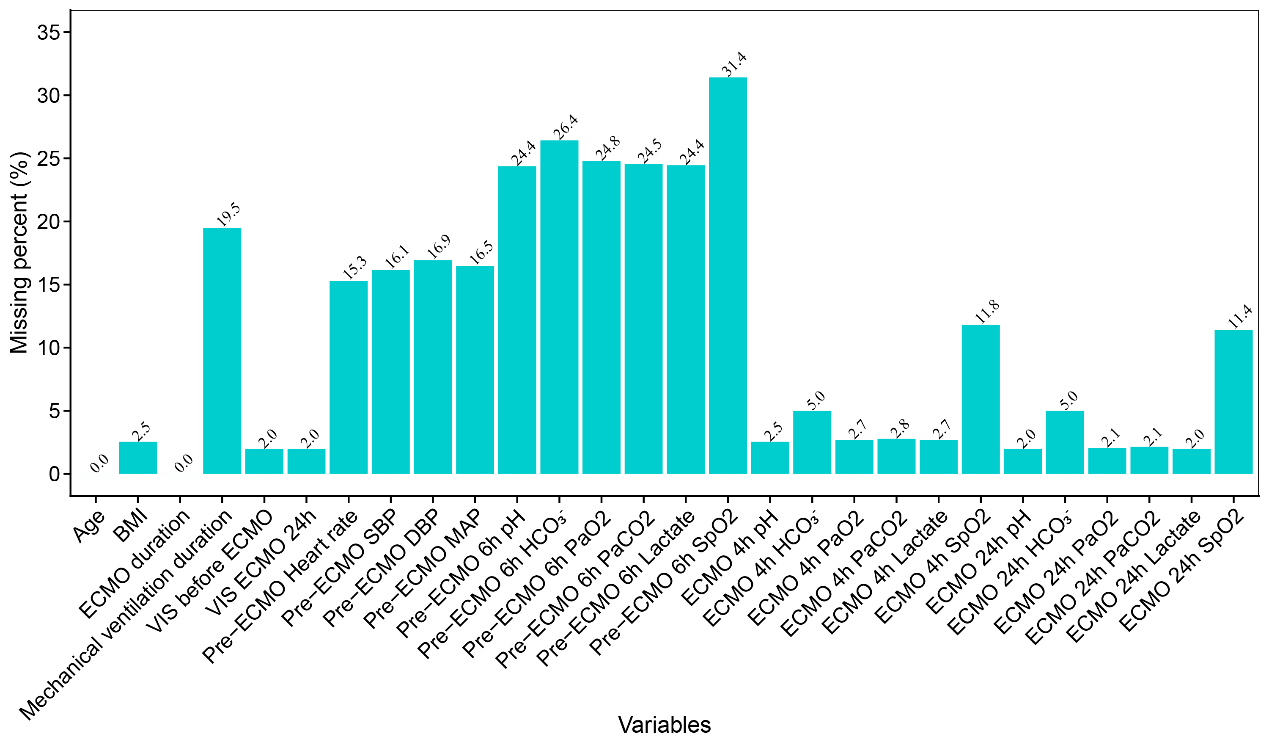
**

**Figure S3. Percentages of missing data for all included variables in study population.**

Abbreviations: BMI, body mass index; ECMO, extracorporeal membrane oxygenation; VIS, vasoactive-inotropic score; SBP, systolic blood pressure; DBP, diastolic blood pressure; MAP, mean arterial pressure.

**
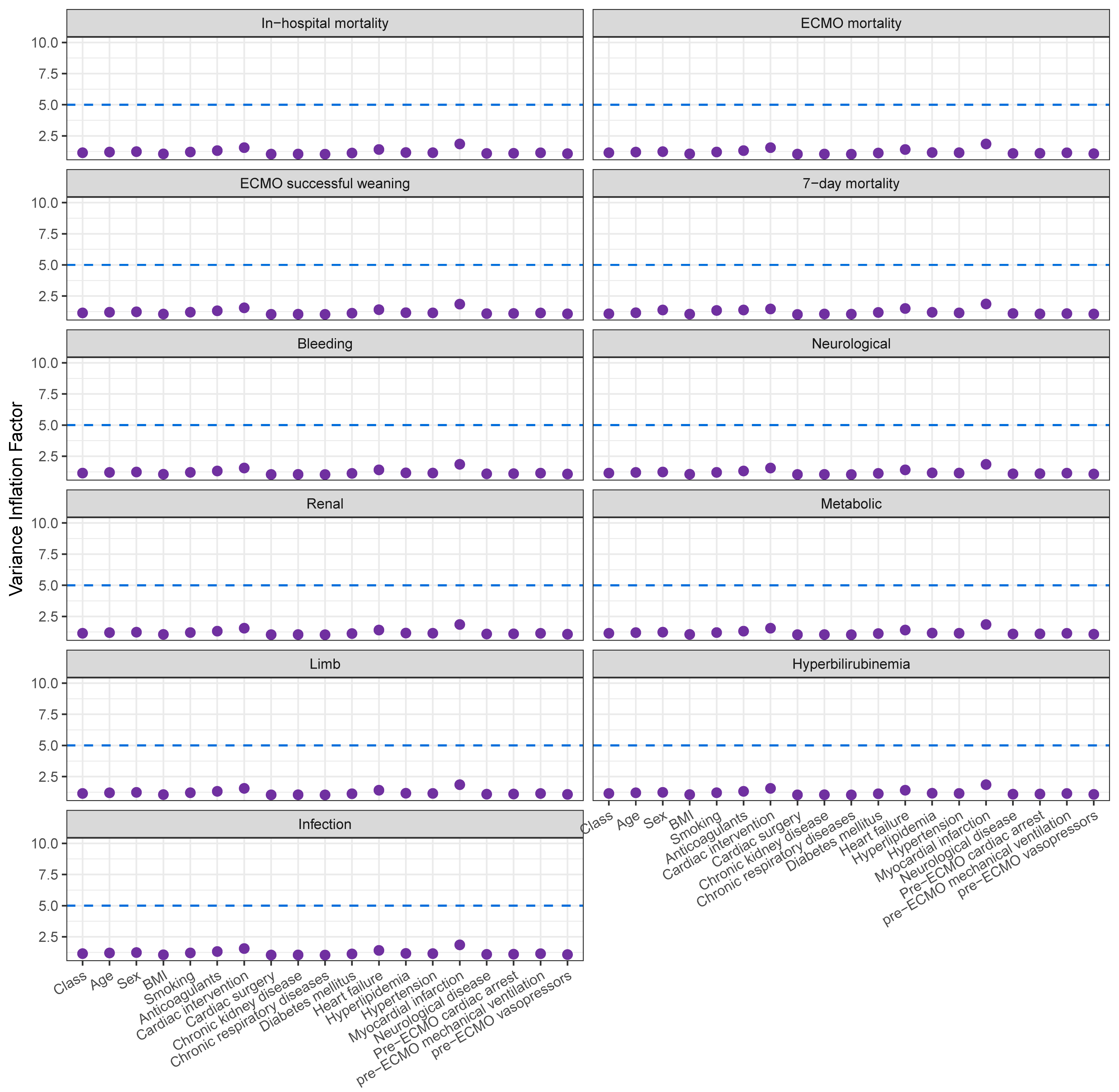
**

**Figure S4. Variance inflation factors for variables included in model 3 for each outcome.** A variance inflation factor < 5 for each variable suggested the absence of multicollinearity. Abbreviations: BMI, body mass index; ECMO, extracorporeal membrane oxygenation.
